# Vesicle-associated Membrane Protein 3 (VAMP3) Mediates Constitutive Trafficking of the Renal Co-transporter NKCC2 in Thick Ascending Limbs: ROLE IN RENAL FUNCTION AND BLOOD PRESSURE*

**DOI:** 10.1074/jbc.M116.735167

**Published:** 2016-08-22

**Authors:** Paulo S. Caceres, Mariela Mendez, Mohammed Z. Haque, Pablo A. Ortiz

**Affiliations:** From the ‡ Hypertension and Vascular Research Division, Department of Internal Medicine, Henry Ford Hospital, Detroit, Michigan 48202,; the § Department of Physiology, Wayne State University, Detroit, Michigan 48202, and; the ¶ Interim Translational Research Institute, Academic Health System, Hamad Medical Corporation, 16060 Doha, Qatar

**Keywords:** epithelial cell, exocytosis, kidney, membrane transport, Na-K-Cl cotransporter (NKCC), renal physiology, SNARE proteins, apical surface, blood pressure, urine excretion

## Abstract

Renal cells of the thick ascending limb (TAL) reabsorb NaCl via the apical Na^+^/K^+^/2Cl^−^ co-transporter NKCC2. Trafficking of NKCC2 to the apical surface regulates NKCC2-mediated NaCl absorption and blood pressure. The molecular mechanisms by which NKCC2 reaches the apical surface and their role in renal function and maintenance of blood pressure are poorly characterized. Here we report that NKCC2 interacts with the vesicle fusion protein VAMP3, and they co-localize at the TAL apical surface. We observed that silencing VAMP3 *in vivo* blocks constitutive NKCC2 exocytic delivery, decreasing the amount of NKCC2 at the TAL apical surface. VAMP3 is not required for cAMP-stimulated NKCC2 exocytic delivery. Additionally, genetic deletion of VAMP3 in mice decreased total expression of NKCC2 in the TAL and lowered blood pressure. Consistent with these results, urinary excretion of water and electrolytes was higher in VAMP3 knock-out mice, which produced more diluted urine. We conclude that VAMP3 interacts with NKCC2 and mediates its constitutive exocytic delivery to the apical surface. Additionally, VAMP3 is required for normal NKCC2 expression, renal function, and blood pressure.

## Introduction

In the kidney, NaCl reabsorption by the thick ascending limb (TAL)^2^ of the loop of Henle is fundamental for concentrating the urine and control of blood pressure. NaCl absorption by the TAL is mediated by the apical Na^+^/K^+^/2Cl^−^ co-transporter NKCC2. Under baseline conditions (absence of external stimuli), NKCC2 is present at the apical surface and also in intracellular vesicles in the subapical space (1). NKCC2 undergoes constitutive exocytic delivery (2, 3), endocytic retrieval, and recycling (4–6) in the absence of any stimulation, maintaining steady-state surface NKCC2 expression at a baseline level. This process of NKCC2 trafficking is a critical determinant of the absorptive capability of the TAL (7–9). Mutagenesis of key amino acids in the carboxyl terminus of NKCC2 results in decreased trafficking to the apical membrane (10). These amino acids are known to be mutated in Bartter syndrome (11), a pathology characterized by loss of NKCC2 function (12–14).

NKCC2 exocytic delivery to the membrane can be stimulated by cAMP. We recently showed that the vesicle-associated membrane protein 2 (VAMP2), of the SNARE family of membrane fusion proteins, mediates a pathway for NKCC2 exocytic delivery that is stimulated by cAMP (3). However, constitutive exocytic delivery of NKCC2 to the plasma membrane was not dependent on VAMP2, indicating that some other VAMP isoform must be involved. We previously showed that VAMP3 is expressed at an apical localization in TALs (9). Thus we hypothesize that constitutive and cAMP-stimulated NKCC2 trafficking are independently controlled by distinct VAMP isoforms and that VAMP3 mediates most of the constitutive NKCC2 exocytic delivery in native renal TALs. Through this mechanism, VAMP3 contributes to steady-state surface NKCC2 expression under physiological conditions and maintains renal NaCl absorption and blood pressure homeostasis.

## Results

### 

#### 

##### Expression of VAMP3 in the Thick Ascending Limb and Interaction with NKCC2

**FIGURE 1.**
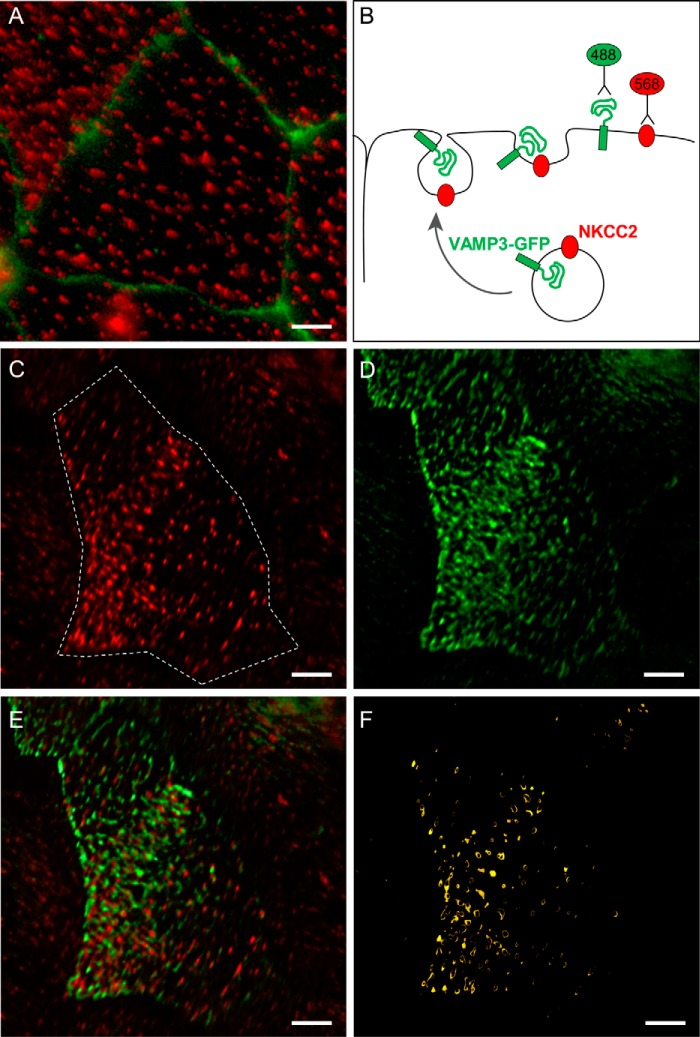
**NKCC2 and VAMP3 co-localize at the apical surface of TALs.** *A*, endogenous expression of NKCC2 in clusters at the apical surface of TAL primary cultures. Apical surface NKCC2 was detected with an antibody against an extracellular epitope applied to the apical side of non-permeabilized cultures (*red*). Individual cells are delimited by the tight junction marker ZO1 (*green*). *B*, schematic representation of the procedure followed to co-localize NKCC2 and VAMP3 at the apical surface of TAL primary cultures. The cells were transduced with VAMP3-GFP, which was detected at the apical surface with an anti-GFP antibody before permeabilizing. Endogenous NKCC2 was detected at the apical surface with the extracellular antibody. *C*, surface NKCC2 expression in TAL primary cultures (single cell indicated by the *dashed line*). *D*, surface VAMP3-GFP expression in apical clusters in TAL primary culture (same cell as in *C*). *E*, merged image showing co-localization of surface NKCC2 and surface VAMP3-GFP as indicated by the *yellow color* in some clusters. *F*, the apical clusters where surface NKCC2 and VAMP3 co-localize were identified by isolating the co-localizing pixels with a Mander's overlap coefficient ≥ 0.95 (*n* = 6). *Scale bar*, 1 μm.

**FIGURE 2.**
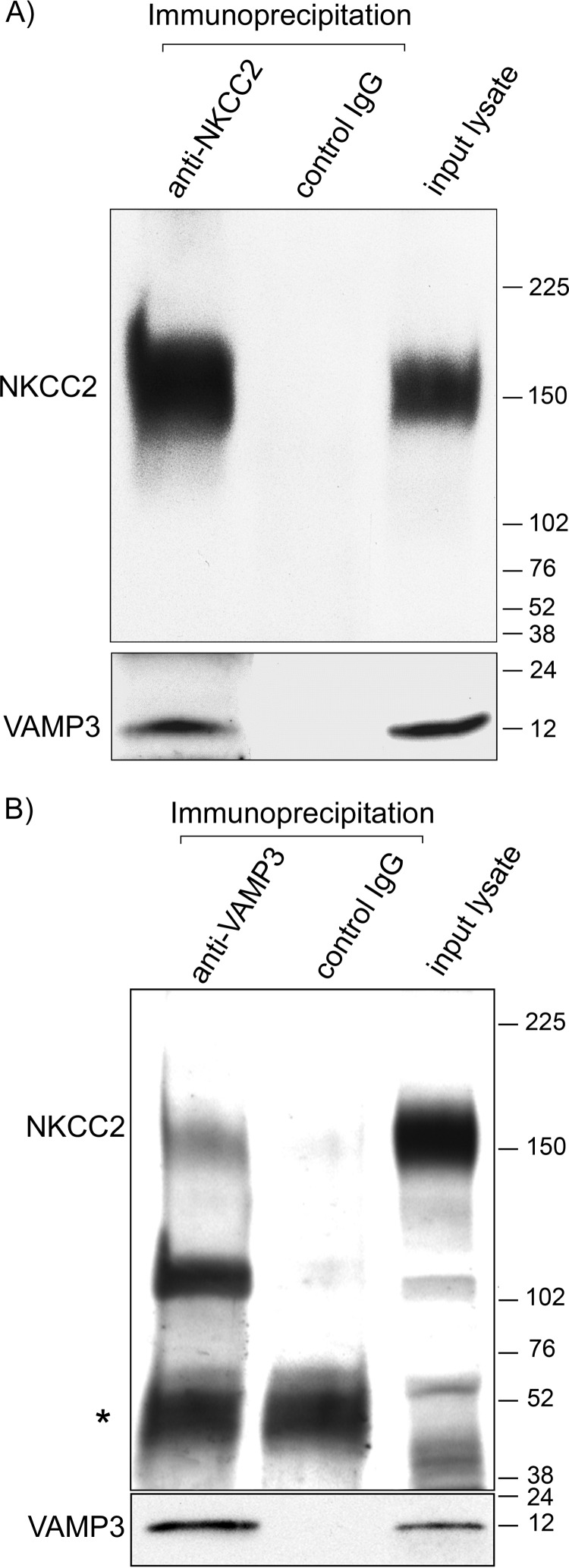
**VAMP3 co-immunoprecipitates with NKCC2 in native TALs.** *A*, endogenous NKCC2 was immunoprecipitated from TAL lysates with a rabbit antibody specific for NKCC2. Co-immunoprecipitation was detected by Western blotting against NKCC2 (chicken IgY) and VAMP3 (rabbit IgG). *B*, reciprocal immunoprecipitation with a rabbit anti-VAMP3 antibody co-immunoprecipitated VAMP3 and NKCC2 in TAL lysates (endogenous proteins detected with rabbit antibodies). Control IgG failed to precipitate VAMP3 or NKCC2. Co-immunoprecipitations were performed from 200 μg of TAL protein lysates, and all the immunoprecipitate obtained from the starting material was loaded in the gel. In the *input lysate lanes*, 5 μg of TAL lysate was loaded. The rabbit antibody against NKCC2 detected a 160-kDa band corresponding to the glycosylated form of NKCC2 and a lower band (∼120 kDa) corresponding to non-glycosylated NKCC2 (64). The rabbit IgG used for the immunoprecipitation was detected by the anti-rabbit secondary antibody (*; *n* = 4).

We and others have shown that VAMP3 is expressed apically in TALs and medullary collecting ducts (9, 15, 16). We recently showed that the related isoform VAMP2 co-localizes with NKCC2 at the apical membrane of TALs (3). Here, we tested whether VAMP3 also co-localizes with NKCC2 at the apical surface of TALs. As we observed previously (3), NKCC2 was localized at discrete clusters in the apical membrane of polarized TAL primary cultures (Fig. 1, *A* and *C*). We transfected the cultures with a VAMP3-GFP construct that, when expressed, faces the GFP tag toward the vesicle lumen (Fig. 1*B*). When the VAMP3-GFP vesicles fuse with the apical membrane, the GFP tag faces the extracellular space. Because VAMP3 does not have an extracellular domain, the GFP tag allowed us to detect surface VAMP3 with an extracellular antibody against GFP. Similar to surface NKCC2, VAMP3-GFP was distributed in apical clusters in TALs (Fig. 1*D*). We observed co-localization between NKCC2 and VAMP3-GFP in apical clusters at the cell surface (Fig. 1, *E* and *F*). To test whether VAMP3 interacts with NKCC2 in native TALs, we performed co-immunoprecipitations in TAL lysates. We observed that VAMP3 co-immunoprecipitated with NKCC2 (Fig. 2*A*), and this was confirmed by the reciprocal co-immunoprecipitation with an anti-VAMP3 antibody (Fig. 2*B*). Altogether, these results indicate that VAMP3 is expressed apically in the TAL, co-localizes with NKCC2 at apical surface clusters, and interacts with NKCC2.

##### Effect of VAMP3 Silencing on Constitutive and cAMP-stimulated Steady-state Surface NKCC2 Expression

**FIGURE 3.**
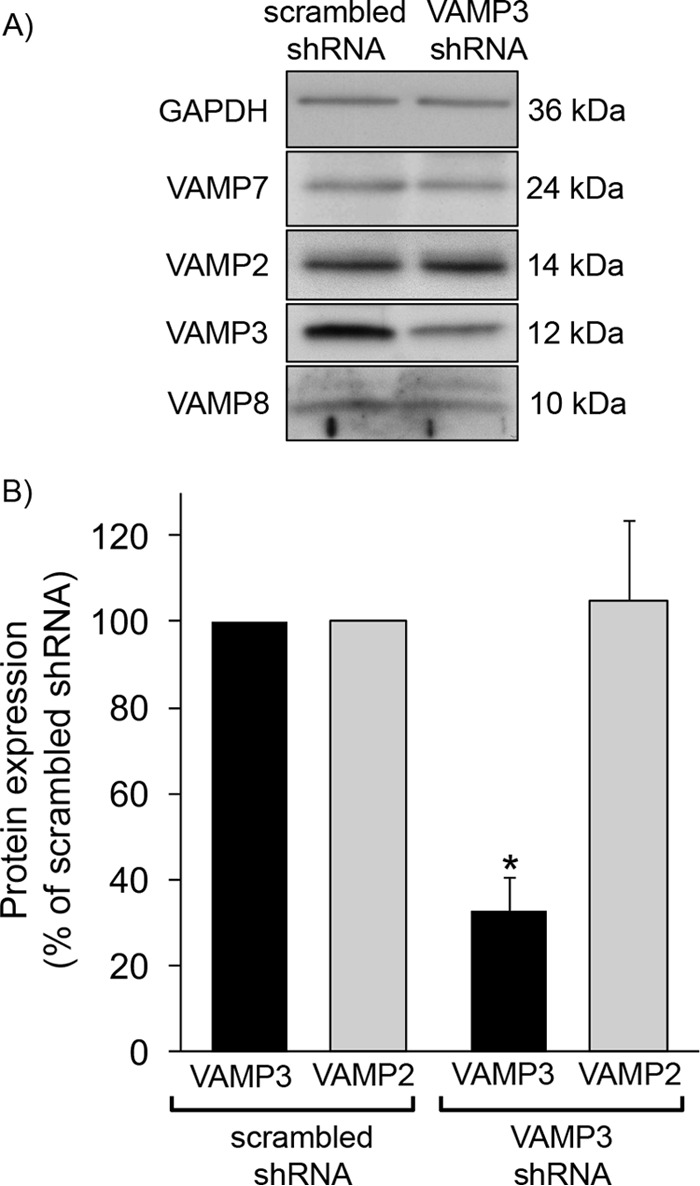
**Silencing of VAMP3 *in vivo* in TALs via shRNA.** *A*, Western blot showing decreased VAMP3 expression in rat TALs after transduction with adenoviruses carrying VAMP3-shRNA but not scrambled-shRNA. Expression of GAPDH or other VAMP isoforms was not affected. *B*, quantification of VAMP3 and VAMP2 expression after silencing VAMP3 *in vivo* in TALs. After 3 days of silencing, VAMP3 expression was 33 ± 7% of scrambled-shRNA, and VAMP2 expression was 105 ± 19% of scrambled-shRNA. The values are expressed as mean percentages of scrambled-shRNA. *Error bars* represent ± S.E. *, *p* < 0.05 *versus* scrambled-shRNA (*n* = 6).

**FIGURE 4.**
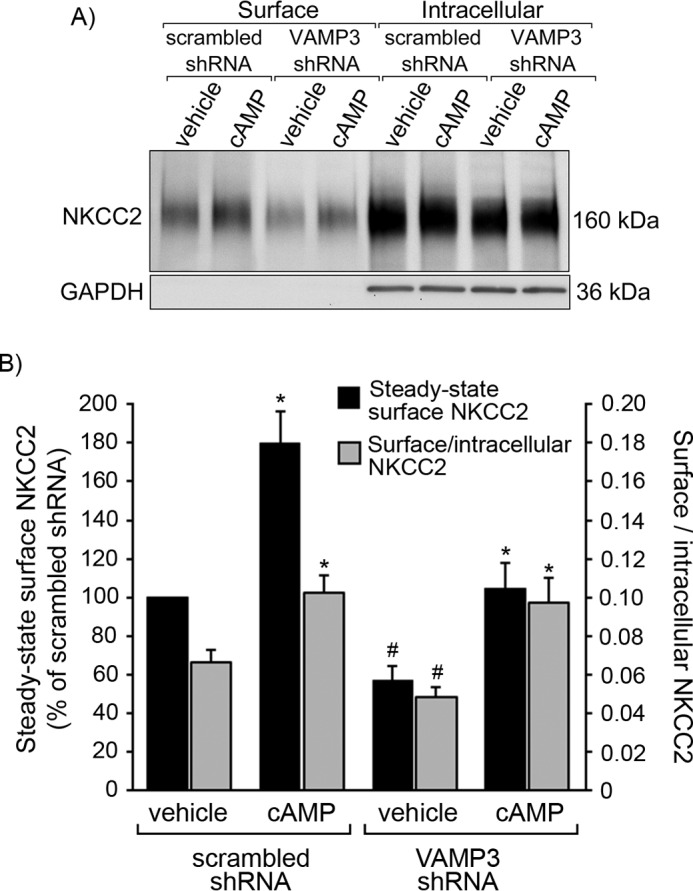
**Silencing VAMP3 decreases constitutive steady-state surface NKCC2 expression in rat TALs.** *A*, representative surface biotinylation experiment showing steady-state surface and intracellular NKCC2 in TALs from rats transduced *in vivo* with VAMP3-shRNA or scrambled-shRNA. TALs were treated with vehicle or forskolin + IBMX to stimulate cAMP. Intracellular protein GAPDH was not detected at the surface. *B*, quantification of steady-state surface NKCC2 after silencing VAMP3 as measured by surface biotinylation in TALs. In VAMP3-shRNA transduced TALs, the constitutive (vehicle) surface to intracellular NKCC2 ratio was decreased by 27% (*gray bars*), and steady-state surface NKCC2 was reduced by 43% ± 7% (*black bars*). However, cAMP increased steady-state surface NKCC2 expression equally in scrambled-shRNA and VAMP3-shRNA-transduced TALs. The values represent the averages in each experimental group. *Error bars* represent ± S.E. *, *p* < 0.05 *versus* vehicle; #, *p* < 0.05 *versus* vehicle/scrambled-shRNA (*n* = 10).

We previously showed that VAMP2 mediates cAMP-stimulated steady-state surface NKCC2 expression but not constitutive surface NKCC2 expression in the TAL (3). To test whether VAMP3 mediates constitutive steady-state surface NKCC2 expression, we silenced VAMP3 in rat TALs. We previously developed shRNA to silence VAMP3 *in vivo* via adenovirus transduction directly to the renal outer medulla (3). To assure that VAMP3 silencing was specific, we measured expression of VAMP3 and other VAMPs in VAMP3-shRNA-transduced TALs and compared with scrambled-shRNA-transduced TALs from the same rat (Fig. 3*A*). We observed that after 3–4 days of silencing, VAMP3 expression was reduced by almost 70% in TALs *in vivo* (Fig. 3*B*). There was no noticeable change in expression of other VAMP isoforms. After we silenced VAMP3, we obtained TAL suspensions, treated them with vehicle (constitutive) or forskolin + IBMX (cAMP-stimulated), and measured surface NKCC2 expression by surface biotinylation (Fig. 4*A*). To assure that intracellular proteins were not biotinylated, we determined that GAPDH was only expressed at the intracellular fraction but not at the surface. This control was also performed in all subsequent surface biotinylation procedures.

We observed that the surface to intracellular NKCC2 ratio was decreased by 27% in TALs when VAMP3 was silenced (scrambled-shRNA: 0.066 ± 0.006 *versus* VAMP3-shRNA: 0.049 ± 0.005, *p* < 0.05; Fig. 4*B*). This reflected in a 43% decrease in constitutive steady-state surface NKCC2 expression (scrambled-shRNA: 100% *versus* VAMP3-shRNA: 57 ± 7%, *p* < 0.05; Fig. 4*B*). Interestingly, stimulation by cAMP was not affected by VAMP3 silencing. cAMP stimulated steady-state surface NKCC2 by 80% in scrambled-shRNA-transduced TALs (from 100% to 180 ± 7%) and by 82% in VAMP3-silenced TALs (from 57 ± 7% to 104 ± 13%). These data indicate that VAMP3 partially mediates constitutive steady-state surface NKCC2 expression but does not mediate cAMP-stimulated surface NKCC2 expression.

##### Effect of VAMP3 Silencing on Constitutive and cAMP-stimulated NKCC2 Exocytic Delivery

**FIGURE 5.**
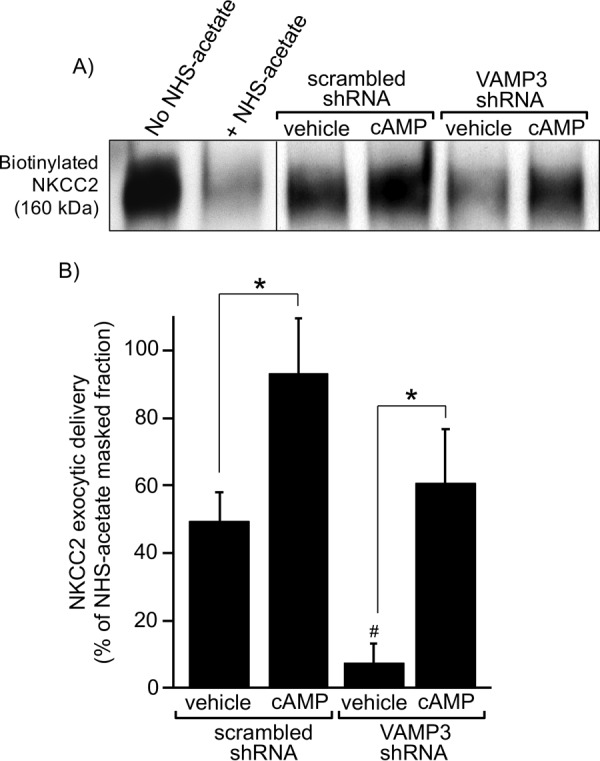
**Silencing VAMP3 blocks constitutive NKCC2 exocytic delivery in rat TALs.** *A*, representative Western blot showing masking of surface biotinylation sites by NHS-acetate at 4 °C and reappearance of surface NKCC2 signal after exocytic delivery at 37 °C in TALs transduced with scrambled or VAMP3-shRNA. TALs were treated with vehicle or forskolin + IBMX to stimulate cAMP. The *vertical division line* separates non-consecutive lanes in the same gel and film. *B*, quantification of NKCC2 exocytic delivery at 30 min in rat TALs measured as biotinylated NKCC2 at the surface after masking with NHS-acetate. In rats transduced *in vivo* with VAMP3-shRNA, constitutive NKCC2 exocytic delivery was decreased by 86% after silencing VAMP3. However, cAMP was still able to stimulate NKCC2 exocytic delivery after silencing VAMP3. For every experiment (scrambled-shRNA and VAMP3-shRNA), the difference between the non-NHS-acetate masked fraction and the NHS-acetate masked fraction at time 0 was used as reference to calculate the NHS-acetate masked fraction. The values represent the mean percentages of NHS-acetate masked fraction. *Error bars* represent ± S.E. *, *p* < 0.05 *versus* vehicle; #, *p* < 0.05 *versus* vehicle/scrambled-shRNA (*n* = 6).

Steady-state surface NKCC2 expression is maintained by a balance between constitutive exocytic delivery, endocytic retrieval, and recycling (2–6, 9) that occurs in the absence of stimuli. To test whether VAMP3 mediates constitutive NKCC2 exocytic delivery, we silenced VAMP3 via shRNAs and measured NKCC2 exocytic delivery. For this, we masked surface biotinylation sites with NHS-acetate, then allowed exocytosis to proceed at 37 °C and we detected newly exocytosed NKCC2 by surface biotinylation as described before (2, 3). We observed that silencing VAMP3 significantly blocked the baseline rate of NKCC2 exocytic delivery compared with control TALs (scrambled-shRNA: 49 ± 9% of NHS-acetate masked fraction *versus* VAMP3-shRNA: 7 ± 6% of NHS-acetate masked fraction, *p* < 0.05) (Fig. 5*B*). However, cAMP strongly stimulated NKCC2 exocytic delivery in TALs transduced with scrambled-shRNA (93 ± 17% of NHS-acetate masked fraction) and VAMP3-shRNA (61 ± 16% of NHS-acetate masked fraction). These data indicate that VAMP3 mediates constitutive NKCC2 exocytic delivery in TALs. However, silencing VAMP3 does not prevent the stimulatory effect of cAMP on the exocytic rate of NKCC2, because this was previously shown to be mediated by VAMP2 (3).

##### Effect of VAMP3 Silencing on Total NKCC2 Expression

**FIGURE 6.**
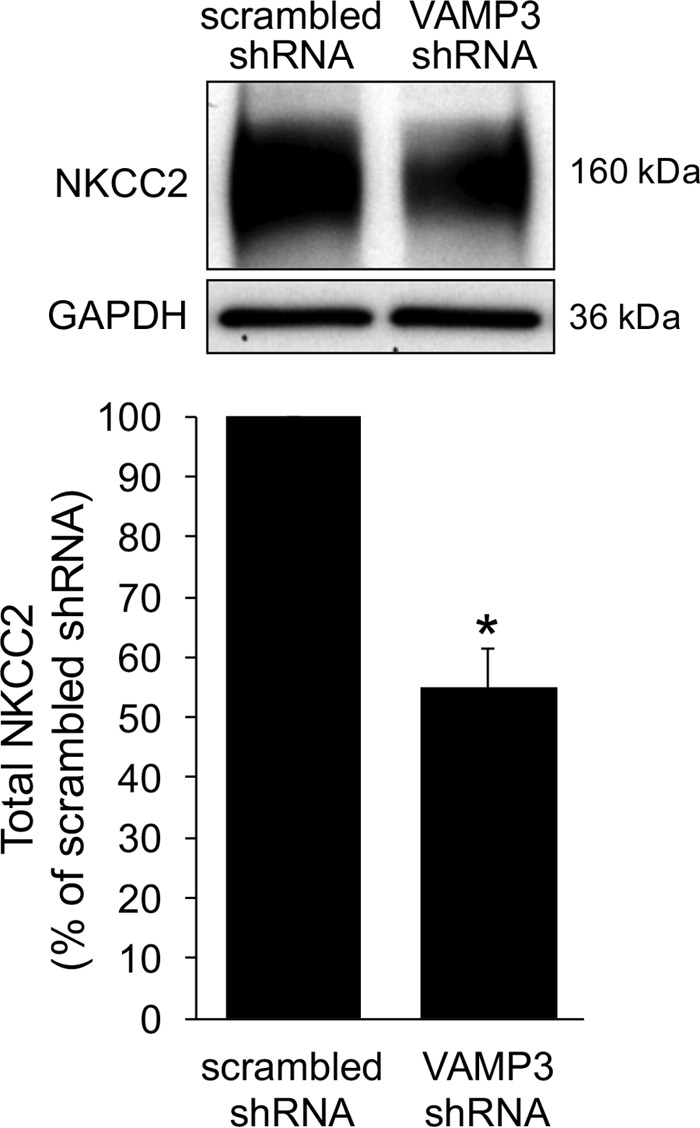
**Silencing VAMP3 decreases total NKCC2 expression.** The rats were transduced *in vivo* with VAMP3-shRNA adenoviruses, and total NKCC2 expression was measured by Western blot. Silencing VAMP3 decreased NKCC2 expression by 45%. The values are expressed as mean percentages of scrambled-shRNA. *Error bars* represent ± S.E. *, *p* < 0.05 *versus* scrambled-shRNA (*n* = 10).

To test whether VAMP3 is required for baseline NKCC2 expression, we silenced VAMP3 in rats for 4 days and measured total NKCC2 expression in TALs. We observed that VAMP3-shRNA decreased total NKCC2 expression by 45% (scrambled-shRNA: 100% *versus* VAMP3-shRNA: 55 ± 6%, *p* < 0.05) (Fig. 6). These observations suggest that VAMP3 is required to maintain normal levels of NKCC2 protein in TALs.

##### Total and Steady-state Surface NKCC2 Expression in VAMP3^−/−^ Mice

**FIGURE 7.**
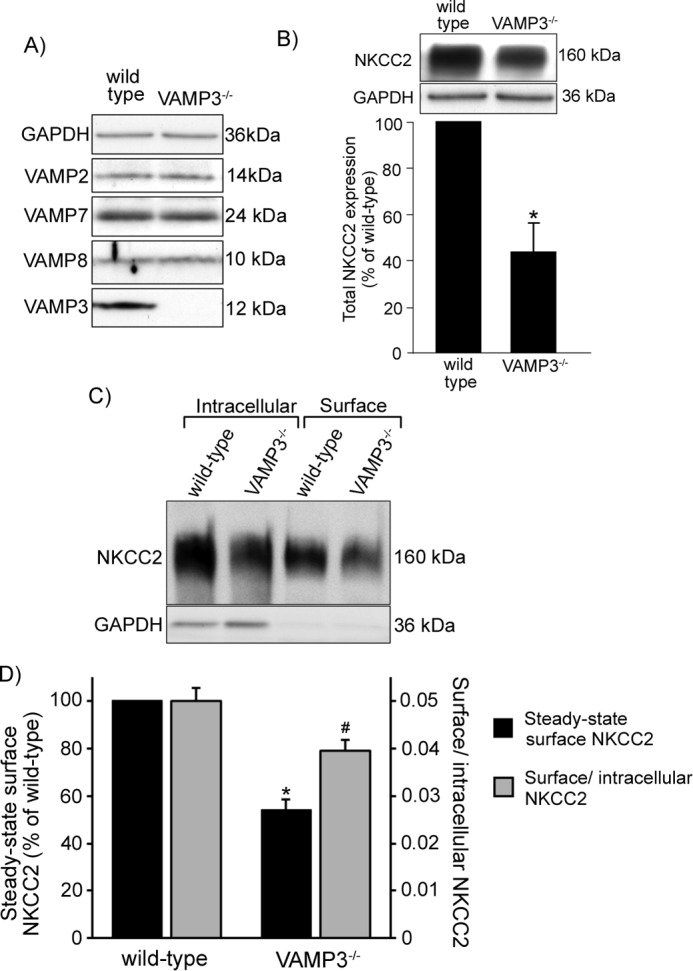
**Decreased total and steady-state surface NKCC2 expression in TALs from VAMP3^−/−^ mice.** *A*, Western blot confirming absence of VAMP3 in TALs from VAMP3^−/−^ mice, whereas expression of other VAMP isoforms remained unchanged. *B*, total NKCC2 expression was measured by Western blot in TALs obtained from wild-type and VAMP3^−/−^ mice. NKCC2 was decreased by 66 ± 12% in VAMP3^−/−^ mice compared with wild type. The values are expressed as mean percentages of wild-type mice. *Error bars* represent ± S.E. *, *p* < 0.05 *versus* wild-type mice. *C*, representative surface biotinylation experiment showing intracellular and steady-state surface NKCC2 expression in TALs from wild-type and VAMP3^−/−^ mice. *D*, quantification of steady-state surface NKCC2 expression in VAMP3^−/−^ mice. Steady-state surface NKCC2 was decreased by 46 ± 4% in VAMP3^−/−^ mice compared with wild type (*black bars*, representing mean percentage of wild-type mice). *Error bars* represent ± S.E. *, *p* < 0.01 *versus* wild type. The surface to intracellular NKCC2 ratio was decreased by 20% (*gray bars*, representing mean surface to intracellular NKCC2 ratio). *Error bars* represent ± S.E. #, *p* = 0.04 *versus* wild type (*n* = 5).

The acute effect of silencing VAMP3 is striking because it decreases total and surface NKCC2 expression by ∼50%. To further study whether this inhibition is maintained chronically and of biological relevance *in vivo*, we studied VAMP3^−/−^ mice. These mice were generated previously by Yang *et al.* (17) and displayed no major abnormal phenotype. However, the renal phenotype of these mice has never been determined. We obtained TAL suspensions from VAMP3^−/−^ mice and confirmed that they do not express VAMP3 (Fig. 7*A*). We did not detect changes in expression of other VAMP isoforms that may compensate for the lack of VAMP3, including VAMP2, VAMP7, or VAMP8. Gross kidney anatomy and morphology appeared normal in adult VAMP3^−/−^ mice as evaluated by macroscopic appearance and histologic analysis of the renal cortex and medulla in hematoxylin- and eosin-stained sections (not shown).

Body and kidney weights were comparable between wild-type and VAMP3^−/−^ mice, as well as plasma Na^+^, plasma K^+^ and food consumption (Table 1). Next, we measured total NKCC2 expression in TALs from VAMP3^−/−^ mice and found it to be decreased by 66 ± 12% (*p* < 0.05) compared with control wild-type mice (Fig. 7*B*). This finding is consistent with our observations from VAMP3 silencing experiments. Because VAMP3 mediates constitutive NKCC2 trafficking, we next tested whether steady-state surface NKCC2 expression was decreased in VAMP3^−/−^ mice (Fig. 7*C*). We measured surface NKCC2 by surface biotinylation in TALs and observed a decrease of 46% in steady-state surface NKCC2 expression compared with wild-type mice (wild type = 100% *versus* VAMP3^−/−^ = 54 ± 4, *p* < 0.01) (Fig. 7*D*). To confirm that this decrease on surface NKCC2 was not just a consequence of decreased NKCC2 expression, we measured the surface to intracellular NKCC2 ratio. We observed that the surface to intracellular NKCC2 ratio was 0.050 ± 0.003 in TALs from wild-type mice but only 0.040 ± 0.002 (a 20% decrease) in VAMP3^−/−^ mice. Altogether these observations confirm that VAMP3 mediates steady-state surface and total NKCC2 expression in the TAL.

**TABLE 1:** **Body weights, kidney weights, plasma ions, and food consumption in wild-type and VAMP3^−/−^ mice** The values are the means ± S.E. The *p* values for comparisons between wild type and VAMP3^−/−^ are indicated. No significant difference was found for all the variables measured (*n* = 5).

	Wild type	VAMP3^−/−^	*p* value
Body weight (g)	28.28 ± 0.97	29.17 ± 0.51	0.44
Left kidney weight (mg)	168 ± 11	172 ± 5	0.74
Right kidney weight (mg)	170 ± 10	182 ± 7	0.36
Plasma Na^+^ (mm)	145.6 ± 0.7	145.0 ± 0.5	0.53
Plasma K^+^ (mm)	5.0 ± 0.2	4.4 ± 0.2	0.09
Normal diet consumed (g/day)	4.4 ± 0.1	4.2 ± 0.2	0.43
24-h low-Na^+^ diet consumed (g/day)	0.8 ± 0.3	0.8 ± 0.3	0.91
48-h low-Na^+^ diet consumed (g/day)	3.6 ± 0.2	3.4 ± 0.1	0.26

##### Role of VAMP3 in Renal Function

Because steady-state surface NKCC2 expression is directly related to TAL NaCl absorption, and this was decreased in VAMP3^−/−^ mice, we next studied whether VAMP3 is required for normal renal NaCl absorption. We placed VAMP3^−/−^ and wild-type mice in metabolic cages and measured urine volume, osmolality, and excretion of Na^+^, K^+^, Cl^−^, Ca^2+^, and Mg^2+^ (Table 2). We observed that VAMP3^−/−^ mice produced a more diluted urine and excreted more Ca^2+^. However, we did not observe any major difference in urine volume or excretion of other ions. TAL NaCl reabsorption is known to be required for maximal urinary concentration and salt absorption. Next we challenged VAMP3^−/−^ to water deprivation and measured how they adjusted urinary excretion. After 24 h of water deprivation, VAMP3^−/−^ mice excreted more Na^+^, Cl^−^, and K^+^ than wild-type mice (Table 2). As expected, water deprivation increased circulating vasopressin in both strains, but we did not detect any significant difference between wild-type and VAMP3^−/−^ mice (Table 2).

**TABLE 2:** **Water consumption, renal parameters, and circulating vasopressin in wild-type and VAMP3^−/−^ mice** Blood and 24-h urine samples were collected to measure volume, osmolality, urinary Na^+^ (UNa^+^), urinary K^+^ (UK^+^), urinary Cl^−^ (UCl^−^), urinary Ca^2+^ (UCa^2+^), urinary Mg^2+^ (UMg^2+^), urinary creatinine (UCreatinine) excretion, and serum vasopressin. The measurements were performed under baseline conditions and after 24-h water deprivation. The values are the means ± S.E. The *p* values for comparisons between wild type and VAMP3^−/−^ are indicated (*n* = 6).

	Baseline			24-h water deprivation		
	Wild type	VAMP3^−/−^	*p* value	Wild type	VAMP3^−/−^	*p* value
Water consumed (ml/24 h)	4.7 ± 0.2	5.1 ± 0.3	0.35			
Urine volume (μl/24 h)	1337 ± 118	1625 ± 167	0.19	650 ± 85	673 ± 85	0.86
Urine osmolality (mOsm)	3433 ± 166	2924 ± 131	0.04*^a^*	5307 ± 235	5763 ± 456	0.40
UNa^+^ (μmol/day)	264.8 ± 18.9	266.3 ± 31.2	0.97	445.3 ± 33.2	591.4 ± 53.8	0.04*^a^*
UK^+^ (μmol/day)	779.8 ± 51.2	781.9 ± 98.2	0.99	1100.0 ± 77.8	1491.3 ± 105.8	0.01*^a^*
UCl^−^ (μmol/day)	541.4 ± 43.7	611.5 ± 65.8	0.40	823.7 ± 57.0	1137.9 ± 92.9	0.02*^a^*
UCa^2+^ (μmol/day)	0.60 ± 0.02	0.81 ± 0.05	0.01*^a^*	1.06 ± 0.12	1.38 ± 0.12	0.08
UMg^2+^ (μmol/day)	1.90 ± 0.19	1.73 ± 0.13	0.50	5.47 ± 0.62	5.16 ± 0.58	0.73
UCreatinine (mg/day)	0.31 ± 0.02	0.35 ± 0.04	0.37	0.89 ± 0.09	1.17 ± 0.12	0.09
Vasopressin (pg/ml)	5.7 ± 1.4	4.6 ± 1.7	0.64	41.9 ± 8.5	64.4 ± 12.1	0.16

*^a^ p* < 0.05.

**FIGURE 8.**
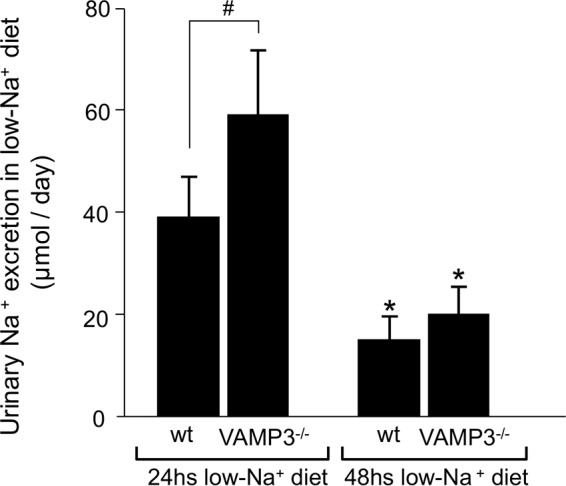
**Delayed adjustment of Na^+^ excretion in response to restricted Na^+^ intake in VAMP3^−/−^ mice.** VAMP3^−/−^ mice fed a low Na^+^ diet (0.02% Na^+^) for 24 h showed higher excretion of Na^+^ in the urine compared with wild type. However, after 48 h on a low Na^+^ diet, Na^+^ excretion was comparable between strains. The values are expressed as mean 24-h urinary Na^+^ excretion. *Error bars* represent ± S.E. *, *p* < 0.05 *versus* 24-h low Na^+^ diet; #, *p* = 0.03 *versus* wild-type mice (*n* = 6).

To study whether the ability to reabsorb Na^+^ is decreased in VAMP3^−/−^ mice, we fed mice a low Na^+^ diet. After 24 h on a low Na^+^ diet, VAMP3^−/−^ mice excreted more Na^+^ than wild type (wild type = 39 ± 8 μmol/day *versus* VAMP3^−/−^ = 59 ± 12 μmol/day, *p* < 0.05) (Fig. 8). However, after 48 h on a low Na^+^ diet, Na^+^ excretion was comparable with that of wild-type mice (wild type = 15 ± 6 μmol/day *versus* VAMP3^−/−^ = 20 ± 8 μmol/day). These data indicate that VAMP3 is required for normal renal Na^+^ absorption under dietary Na^+^ restriction, and VAMP3^−/−^ mice take longer (48 h) to adjust Na^+^ excretion compared with wild-type mice.

##### Effect of Genetic Deletion of VAMP3 in Blood Pressure

**FIGURE 9.**
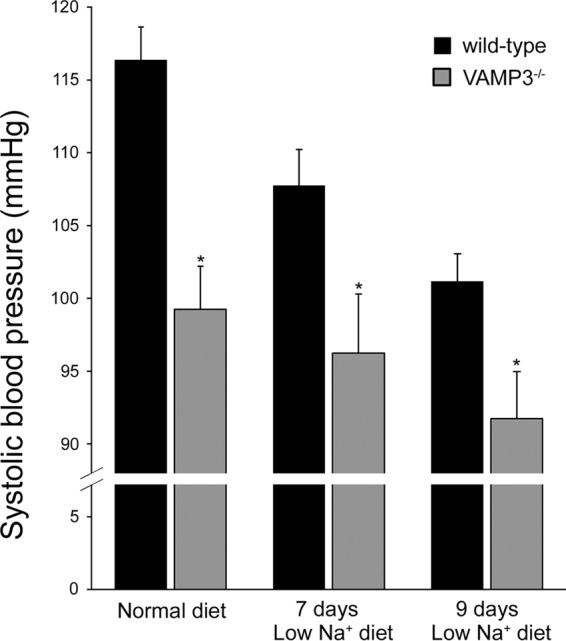
**Decreased blood pressure in VAMP3^−/−^ mice.** Blood pressure was measured by tail cuff in wild-type and VAMP3^−/−^ mice. Systolic blood pressure was lower in VAMP3^−/−^ mice, and this difference was maintained after 7 and 9 days on a low Na^+^ diet. The values are expressed as mean systolic blood pressure. *Error bars* represent ± S.E. *, *p* < 0.05 *versus* wild type (*n* = 6).

Enhanced Na^+^ excretion during low Na^+^ diet indicates that Na^+^ homeostasis is affected in VAMP3^−/−^ mice. If Na^+^ is not appropriately retained, blood pressure is expected to be reduced in VAMP3^−/−^ mice. To study this, we measured systolic arterial pressure in VAMP3^−/−^ mice. In mice fed a normal salt diet, we observed that systolic blood pressure was 19 mm Hg lower in VAMP3^−/−^ mice compared with wild-type mice (wild type = 114 ± 2 mm Hg *versus* VAMP3^−/−^ = 95 ± 2 mm Hg, *p* < 0.05) and remained lower after 7 and 9 days on a low Na^+^ diet (Fig. 9). These results indicate that through the regulation of NKCC2 and renal NaCl absorption, VAMP3 is required to maintain normal blood pressure in mice.

## Discussion

In the rat TAL, most NKCC2 is at intracellular subapical vesicles (1), whereas 3–5% is at the cell surface (9). This small fraction of surface NKCC2 mediates NaCl absorption by the TAL and contributes to the control of blood pressure. We showed that VAMP2, a member of the SNARE family of vesicle fusion proteins, mediates a pathway of NKCC2 exocytic delivery that is stimulated by cAMP (3). However, silencing VAMP2 did not decrease constitutive NKCC2 exocytic delivery in the absence of stimulation. This suggests that the constitutive pathway is controlled by a different SNARE. Here, we report that VAMP3 mediates constitutive NKCC2 delivery and surface NKCC2 expression in the TAL without affecting the cAMP-stimulated pathway.

We observed that VAMP3 and NKCC2 co-immunoprecipitated in TALs and co-localized at discrete clusters in the apical surface. This pattern is similar to the VAMP2-NKCC2 interaction we previously described (3). However, it is not clear from our experiments whether NKCC2 is physically segregated into two distinct VAMP2- and VAMP3-positive pools. In the renin-secreting cells of the juxtaglomerular apparatus, renin granules also express VAMP2, but they exclude VAMP3 (18). Additional reports point toward separate functional pools of exocytic vesicles in other cells. In neurons, the spontaneous release pool and the evoked release pool are constituted by two separate vesicle populations (19, 20). Other reports are discrepant (21–23), but this is likely due to differences in neuronal cell types. In adipose cells, GLUT4 co-localizes with VAMP2 and VAMP3 in different vesicle populations (24). Only the VAMP2-regulated GLUT4 pool is stimulated by insulin (25–28), whereas VAMP3 may mediate GLUT4 recycling (24). VAMP3 has often been associated with constitutive recycling (24, 29–31). NKCC2 undergoes constitutive recycling in the TAL (4). However, further studies are required to determine whether VAMP3 mediates this.

It is striking from our experiments that silencing VAMP3 almost completely blocked NKCC2 exocytic delivery, whereas it only partially decreased steady-state surface NKCC2. This remaining NKCC2 at the TAL surface could be explained by the presence of additional VAMPs in the TAL, such as VAMP7 and VAMP8 (3), which may compensate for the lack of VAMP3, as shown in other cell systems (32–35). Alternatively, the small amount of NKCC2 that reaches the surface could be retained at the plasma membrane by decreased endocytosis, as we have shown occurs after inhibiting endocytosis (4). VAMP7 and VAMP8 may mediate endocytosis because they participate in endosome function in other cells (36, 37). However, to our knowledge, their role in TALs has not been studied.

We observed that VAMP3 is required for normal NKCC2 expression. There are at least two possibilities for this. First, VAMP3 may be required for the formation of NKCC2-carrying vesicles. Initially, VAMP7 (36) and VAMP8 (37), but not VAMP3 (38), were known to participate in intracellular endosome fusion. It was later shown that VAMP3 mediates vesicle-vesicle fusion processes in the endosomal pathway (39–42). We cannot discard this possibility in TALs based on our experimental design. A second alternative is that decreasing VAMP3 expression prevents NKCC2 from reaching the plasma membrane, causing it to be redirected for degradation. Others have shown that sorting NKCC2 to the incorrect location is detrimental for NKCC2-mediated NaCl transport (10, 43). Thus the second alternative is likely because when we silenced VAMP3, NKCC2 exocytic delivery was almost completely blocked. It is worth comparing this with only a 50% decrease on steady-state surface expression, which is not an indirect consequence of a proportional 50% reduction in total protein expression. If that was the case, we would expect a proportional decrease in exocytic delivery instead of complete inhibition. These data indicate that VAMP3 effectively mediates NKCC2 trafficking to the apical membrane rather than simply regulating NKCC2 expression.

Consistent with a role of VAMP3 on NKCC2 trafficking and TAL physiology, we observed that VAMP3^−/−^ mice produced a more dilute urine compared with wild-type mice, and they excreted more Ca^2+^. This is in agreement with the phenotype observed in NKCC2^−/−^ mice (44) and in patients with Bartter syndrome (11, 45–51). However, we did not observe major loss of Na^+^ or K^+^ in urine under baseline conditions. Fluid and Ca^2+^ loss are expected to increase circulating vasopressin and parathyroid hormone, activating the cAMP pathway in the TAL, which is controlled by VAMP2 (3). However, we found no differences in vasopressin between wild-type and VAMP3^−/−^ mice, suggesting that fluid loss is not strong enough to trigger vasopressin release. Our data do not rule out the possibility that other nephron segments distal to the TAL enhance Na^+^, K^+^, and Cl^−^ transport to counteract a major defect in the TAL. This could be mediated by the Na^+^/Cl^−^ co-transporter NCC in the distal tubule (52, 53) or the epithelial Na^+^ channel ENaC in the collecting duct (54, 55).

NKCC2 contributes to urine concentration. To study the maximal ability to concentrate urine or retain Na^+^, we challenged VAMP3^−/−^ mice to water deprivation or low Na^+^ diet. In both cases we observed that VAMP3^−/−^ mice excreted more ions in the urine and took longer to adjust renal excretion. Under water deprivation, VAMP3^−/−^ mice were able to increase circulating vasopressin, reduce urine volume, and concentrate the urine to the same extent as wild-type mice (Table 2). This indicates that the hormonal response to water deprivation is not dependent on VAMP3. However, under this condition we observed increased excretion of Na^+^, K^+^, and Cl^−^, indicating renal wasting of these ions. When mice were placed on a low Na^+^ diet, initially we observed a marked reduction in food intake after 24 h, most likely because of the food being less palatable to mice, because we observed this in both strains and food consumption recovered after 48 h (Table 1). Because we did not detect any difference in food intake or water consumption between strains, we expect a very similar food and water content in the digestive system. We cannot rule out differences in ion absorption by the digestive system because we did not measure this directly. However, our combined data indicate that VAMP3^−/−^ mice have a decreased ability to concentrate their urine and properly excrete electrolytes under baseline conditions and after challenges that demand homeostatic adjustments of water an ion balance. The Na^+^ wasting phenotype is consistent with the lower blood pressure we observed, similar to NKCC2 mutations in humans that also decrease blood pressure (11, 12, 43, 45, 47, 48, 56). However, the possible contribution of other nephron segments and arterial tone should also be considered, although this falls beyond the scope of the hypothesis tested here.

VAMP3^−/−^ mice have been used to study constitutive and insulin-stimulated trafficking (17), granule content release from platelets (57), and phagocytosis by macrophages (58). All of these functions are normal, and the contribution of VAMP3 to whole animal renal function and blood pressure has never been studied. However, the VAMP8 isoform has been studied in the context of whole animal renal physiology (59). VAMP8^−/−^ mice displayed a renal defect that was attributed to inefficient water handling by the collecting duct, but other nephron segments were not studied. The phenotype of VAMP8^−/−^ mice was more severe than what we observed in VAMP3^−/−^ mice. Most likely this depends on the roles different SNAREs play in the regulation of renal transporters in different nephron segments. In this regard, unlike TALs, in collecting duct cells constitutive and cAMP-stimulated exocytosis seem to be equally dependent on VAMP2 and VAMP3 (60, 61).

Our findings in VAMP3^−/−^ mice emphasize the importance of VAMPs in the ability of the kidney to reabsorb water and ions. Further characterization of the mechanism by which VAMP3 mediates NKCC2 exocytic delivery is important to understand the process of renal excretion of water and ions and maintenance of blood pressure.

## Experimental Procedures

### 

#### 

##### Animals

All procedures involving live animals were approved by and conducted following the guidelines from the Institutional Animal Care and Use Committee. Male Sprague-Dawley rats weighing 130 g (Charles River Breeding Laboratories, Wilmington, MA) were used for *in vivo* adenovirus transduction experiments. VAMP3^−/−^ mice were generated previously (17) on a C57BL/6J background. For our experiments we used 9-week-old males and age-matched C57BL/6J as wild-type controls (The Jackson Laboratory, Bar Harbor, ME). Animals were fed a standard diet (0.4% Na^+^, 1% K^+^) or a low sodium diet (0.02% Na^+^, 0.8% K^+^), both from Envigo (Indianapolis, IN).

##### Drugs and Reagents

Reagents for steady-state surface biotinylation and exocytic insertion protocols were from Thermo Fisher Scientific (Waltham, MA). Forskolin and IBMX were from Sigma-Aldrich. Vasopressin was measured with the Arg^8^-Vasopressin ELISA kit from Enzo Life Sciences (Farmingdale, NY) following the manufacturer's specifications.

##### In Vivo Gene Silencing

The target sequence for VAMP3 silencing was a 19-nucleotide sequence from the rat gene: 5′-GGATCTTCTTCGAGACTTT-3′. The sense and antisense sequences, spaced by a loop sequence (TTCAAGAGA), were subcloned between the 5′ AflII and 3′ SpeI sites in the Adenovector-pMIGHTY (Viraquest, North Liberty, IA) for production of adenoviral particles as recently described (3). The adenoviruses were tested in NRK-52E cells at 100 pfu/cell. Expression of VAMP3 and GAPDH was monitored by Western blot. Next, rats were transduced *in vivo* into the outer medulla as described before (3, 5, 62).

##### Suspensions of Medullary TALs

Kidneys were obtained from adenovirus-transduced rats, keeping the left (silenced) and right (control) kidneys separated. TAL suspensions were obtained as described previously (9). Briefly, the outer medulla was dissected, minced, and digested in 0.1% collagenase for 30 min at 37 °C, followed by gentle stirring on ice for 30 min. Finally, TAL suspensions were filtered through a 250-μm nylon mesh.

##### Steady-state Surface NKCC2 Biotinylation of TAL Suspensions

Biotinylation of TAL surface NKCC2 was performed as described before in detail (7, 9). TALs were equilibrated at 37 °C for 15 min and then treated with either vehicle or forskolin (20 μm) plus IBMX (0.5 mm) for 30 min. TALs were biotinylated at 4 °C with NHS-SS-biotin (0.9 mg/ml), washed, and lysed in buffer containing 150 mm NaCl, 50 mm HEPES (pH 7.5), 5 mm EDTA, 1% Triton X-100, 0.2% SDS, and proteases inhibitors. Biotinylated proteins were separated with streptavidin-coated beads overnight at 4 °C, and recovered by boiling in Laemmli loading buffer containing DTT and β-MeEtOH. Proteins were resolved in 6% SDS-polyacrylamide gels, and NKCC2 and GAPDH were detected by Western blot. In every experiment, we monitored total NKCC2 expression and absence of intracellular biotinylation by examination of GAPDH expression in the surface fraction. NKCC2 was detected with chicken anti-rat NKCC2 (1:1400) (raised against an amino-terminal sequence of rat NKCC2) (63) and GAPDH with a monoclonal antibody (1:50,000) from Chemicon (Temecula, CA). Bands were detected by chemiluminescence and quantified.

##### Exocytic Delivery of NKCC2

Surface proteins accessible to NHS-SS-biotin in TAL suspensions were first masked by reaction with membrane-impermeant NHS-acetate as described previously (2, 3). Briefly, TALs were incubated with 2 mg/ml NHS-acetate at 4 °C for 1 h (pH 7.8), adding fresh NHS-acetate every 15 min. Forskolin and IBMX were added at 4 °C, and samples were warmed to 37 °C for 30 min. TALs were then cooled, and newly inserted NKCC2 was biotinylated with NHS-SS-biotin. The efficiency of NHS-acetate masking for NKCC2 was calculated in every experiment (scrambled-shRNA and VAMP3-shRNA-transduced TALs) from the difference between a TAL aliquot that was not masked with NHS-acetate (100% basal surface NKCC2) and an aliquot that was masked at 4 °C but never warmed to 37 °C (0 time point). The difference between these two samples represents the NHS-acetate-masked surface NKCC2 fraction, which was used as reference to express exocytic delivery over time.

##### Co-immunoprecipitation

TAL protein samples were obtained from suspensions lysed in buffer containing 150 mm NaCl, 50 mm HEPES (pH 7.5), 5 mm EDTA, 1% Triton X-100, 0.1% SDS, proteases, and phosphatases inhibitors from Roche Applied Science (Indianapolis, IN). 200 μg of protein from TALs were precleared 30 min at 4 °C with protein G/protein A-coupled agarose beads (Thermo Fisher Scientific) in immunoprecipitation buffer containing 100 mm NaCl, 50 mm HEPES (pH 7.5), 5 mm EDTA, 1% Triton X-100, 1% CHAPS, and proteases inhibitors. Beads were precipitated by centrifugation and discarded. Supernatants were incubated with 5 μg of rabbit IgG against endogenous NKCC2 (3) overnight at 4 °C. Control tubes were incubated with a non-immune rabbit IgG. The next day, protein G/protein A-coupled agarose beads were added in two sequential rounds for a total of 4 h at 4 °C. At the end of the incubation period, beads were sequentially washed in immunoprecipitation buffer, high (500 mm) Na^+^ HEPES, and no Na^+^ HEPES buffer. Proteins were extracted by incubation at 37 °C in loading buffer and run by SDS-polyacrylamide gel electrophoresis (6% gel for NKCC2 and 12% gel for VAMP3). Proteins were transferred to PVDF membranes and blocked in 5% milk in TBS-T buffer for 1 h at room temperature. Next, endogenous NKCC2 and VAMP3 were detected with primary chicken (1:1400 dilution) and rabbit (1:5000 dilution) antibodies, respectively, for 1 h at room temperature. Finally, membranes were incubated with HRP-conjugated secondary antibodies (anti-chicken 1:7000 dilution and anti-rabbit 1:5000 dilution respectively) for 1 h at room temperature and developed by chemiluminescence.

For VAMP3 immuno-precipitation, we used 100 μg of protein lysate and 5 μg of rabbit IgG against endogenous VAMP3 or non-immune rabbit IgG. Proteins were extracted and run by SDS-polyacrylamide gel electrophoresis. Endogenous NKCC2 and VAMP3 were detected by Western blot with rabbit antibodies (1:10,000 and 1:5000 dilutions, respectively).

##### Quantification of Proteins by Densitometry

Mean optical densities for the proteins of interest were measured from x-ray films used for Western blot as routinely performed in our laboratory (9). Films were scanned at 600-dpi resolution, 16-bit grayscale, with an Epson 1680 Expression Pro scanner on positive film mode and saved as uncompressed TIFF. We used an optical density software specifically written to quantify band intensity produced by software engineers at Henry Ford Hospital. Software was calibrated on transmittance mode and used to obtain the mean optical band densities within regions of interest encompassing each individual band in the blot. Background signal was subtracted from an empty region in the same film. The amount of protein loaded was optimized, and several exposure times were quantified to assure that optical densities were within the linear range and not saturated.

##### Co-localization at the Apical Surface of TALs

Primary cultures of TALs were obtained as described before (3). The cells were grown in Transwell® permeable supports (Corning, Tewksbury, MA) coated with basement membrane extract (Trevigen, Gaithersburg, MD). The cells were grown in DMEM supplemented with 1% fetal bovine serum and insulin-transferrin-selenium (Life Technologies) at 37 °C and 5% CO_2_. Cultured TAL cells were transfected with adenoviruses carrying VAMP3-eGFP under control of a CMV promoter for 24 h. The VAMP3-eGFP construct was generously provided by Dr. Romano Regazzi and characterized elsewhere (26). Protein trafficking was rapidly stopped by adding cold medium and incubating the cultures at 4 °C for 30 min. The cells were blocked with 5% albumin in physiological solution for 30 min at 4 °C. Then a primary rabbit antibody against an exofacial epitope on NKCC2 (3, 7) was added to the apical bath (1:50) at 4 °C for 2 h. The cells were washed and incubated with Alexa Fluor 568-conjugated secondary anti-rabbit IgG (1:100) for 1 h at 4 °C. After washout, surface VAMP3-eGFP was labeled with Alexa Fluor 488-conjugated anti-GFP antibody (1:100) for 1 h at 4 °C. Finally, the cells were fixed in 4% paraformaldehyde and mounted. Tight junctions were detected after fixation by labeling with a rabbit antibody against ZO-1 (Zymed Laboratories Inc., Thermo Fisher Scientific) at 1:100 dilution for 1 h at 4 °C and secondary Alexa Fluor 488-conjugated anti-rabbit antibody (1:100 dilution, 1 h at 4 °C). Images were acquired using a laser scanning confocal microscopy system (Visitech, Sutherland, UK) with a 488-nm diode or Kr/Ar 568-nm gas laser excitation controlled by an acousto-optic tunable filter. Images were acquired at 100× (1.4 NA) and fluorescence observed using 525/55-nm Band pass or 590-nm low pass filters, respectively. Image files (TIFF format) were minimally deconvolved with Autoquant software (Media Cybernetics, MD) using two-dimensional blind deconvolution. Images from both channels were aligned, and pixel by pixel co-localization was measured using a Mander's overlap coefficient ≥0.95. An image for overlapping pixels was generated.

##### Blood Pressure Measurements

Systolic arterial pressure was measured by tail cuff in pretrained mice with the MC4000 multichannel blood pressure analysis system. The mice were trained for 2 weeks by performing sham measurements before the beginning of the experiments. Blood pressure was monitored, while the animals were on either a regular diet or a low Na^+^ diet. The mice were placed in a temperature-controlled plate at 36 °C-38 °C, and blood pressure was measured on cycles of 30 s spaced by 3-s intervals. Three preliminary cycles were performed, and blood pressure was measured as the average of the next 10 consecutive cycles.

##### Measurements of Urine Parameters and Blood Ions

The mice were placed in metabolic cages (Techniplast, Exton, PA) with free access to food and water. After a period of 3 days of acclimatization, urine samples were collected for 24 h. We measured urine volume and urine osmolality by freezing point depression with an Advanced model 3300 micro-osmometer (Advanced Instruments Inc., Norwood, MA). We also measured urinary Na^+^, K^+^, Cl^−^, Mg^2+^, Ca^2+^, and creatinine with a Stat Profile pHOx Ultra Analyzer (Nova Biomedical, Waltham, MA). Then mice were restricted from access to water for 24 h. During this period, urine was collected to measure the parameters again. Then we let the mice recover for 3 days, placed them on low Na^+^ diet, and collected urine to measure the parameters again. Serum Na^+^ and K^+^ were measured in a NOVA 1+ analyzer (Nova Biomedical).

##### Statistical Analysis

The results are expressed as means ± S.E. One-way analysis of variance was used to determine differences between means in treatments with more than two groups. Post hoc analysis was performed when differences between means were found using the Bonferroni correction for multiple comparisons. For comparisons between two groups, we used Student's *t* test and paired *t* test for paired samples. *p* < 0.05 was considered statistically significant.

## Author Contributions

P. A. O. conceived and coordinated the study and wrote the paper. P. S. C. wrote the paper, prepared the figures and tables, designed and performed the experiments shown in Figs. 1–7 and in Tables 1 and 2, and analyzed the results. M. Z. H. performed and analyzed the experiments shown in Figs. 8 and 9. M. M. designed, produced, and characterized the adenoviruses for silencing shRNAs, revised the draft, and provided intellectual input. All authors reviewed the results and approved the final version of the manuscript.

## Footnotes

2 The abbreviations used are:

TAL: thick ascending limb
NKCC2: Na^+^-K^+^-2Cl^−^ co-transporter 2
VAMP: vesicle-associated membrane fusion protein
eGFP: enhanced green fluorescent protein
IBMX: 3-isobutyl-1-methylxanthine
VAMP3^−/−^: VAMP3 knockout.
